# Respiratory Impairment and Personal Respirable Dust Exposure among the Underground and Open Cast Gold Miners in Tanzania

**DOI:** 10.29024/aogh.2323

**Published:** 2018-08-31

**Authors:** Matilda Rusibamayila, Eugene Meshi, Simon Mamuya

**Affiliations:** 1 Department of Epidemiology, Dodoma Regional Referral Hospital, Dodoma, TZ; 2 Department of Public Health, University of Dodoma, Dodoma, TZ; 3 Department of Environmental and Occupational Health, Muhimbili University of Health and Allied Sciences, Dar es Salaam, TZ

## Abstract

**Background:**

Mining is one of the most hazardous sectors to work in because it predisposes workers to various hazards including dust. Exposure to dust is inevitable in the mines because the process of extracting gold involves breaking rocks. This dust can penetrate up to the alveoli of the pulmonary system and cause respiratory impairment.

**Objectives:**

To determine respiratory impairment, personal respirable dust exposure levels and associated factors among miners in a gold mine in Tanzania.

**Methods:**

Cross-sectional study design, employing questionnaire, was used for data collection on respiratory symptoms. Lung functions were measured using spirometry. Personal respirable dust exposure was collected from similar exposure groups using air sampling pumps. A simple random sampling technique was used to select 112 participants of the study. Data analysis was done using SPSS computer software version 20.0.

**Results:**

The overall geometric mean (GM) of respirable dust was 0.26 mg/m^3^ (GSD = 0.32) over a mean sampling time of 8 hours (with a range between 7–11 hours). The GM of respirable dust for underground workers was significantly higher (0.41 ± 0.28 mg/m^3^) compared to the open pit workers (0.17 ± 0.23 mg/m^3^) with p < 0.01. For underground workers, the GM of respirable dust was the highest among the bogger operators at 0.53 mg/m^3^ (GSD = 0.27). For open pit workers, the highest GM of respirable dust was found among the quality controllers at 0.39 mg/m^3^ (GSD = 0.18). Respiratory symptoms were phlegm (49.1%), breathlessness (42.9%), cough (37.5%), wheezing (18.8%) and chest tightness (10.7%). Cigarette smokers were more likely to suffer from breathlessness than nonsmokers. The prevalence of airflow obstruction (FEV1/FVC < 0.7) was 1.9%; whereas, the prevalence of lung restriction was 8.8%. The study established that age, smoking habit and previous exposure to dust could not predict lung function impairment.

**Conclusion:**

Despite levels of respirable dust exposure being below the recommended occupational exposure limits, the prevalence of respiratory symptoms was still found to be high among the studied gold miners. This calls for a need to conduct further studies on quartz content of the respirable dust.

## Introduction

Each day approximately 6300 people die from occupational accidents or work-related diseases, totaling 2.3 million deaths annually [1]. It is estimated that 4% of the world’s GDP is lost due to absence from work, treatment costs, disability, compensation and deaths arising from work-related risks [1]. These occupational illnesses, injuries and deaths affect developing countries more than others due to a lack of adequate technical and economic capacity to reach occupational health and safety (OHS) standards.

Because Tanzania is rich in various minerals, such as tanzanite, diamond, coal and gold, it is a sector that provides employment to many [2]. According to an investment benefit study report of 2011, the proportion of nationals employed in mining companies in Tanzania is around 94% [3]. In Tanzania, the mining sector contributes to the fatality rate by 20.53%, exceeded only by the construction (23.73%) and transport (20.61%) sectors [4].

Occupational hazards that miners are exposed to include physical, chemical, biological, ergonomic and psychosocial [5]. Short-term and long-term exposure to dust cause respiratory health problems ranging from acute to chronic [6]. Health consequences that can result from dust exposure include chronic bronchitis, silicosis, tuberculosis, emphysema, renal failure and cancer [7].

According to the American Conference of Governmental Industrial Hygienists (ACGIH), the International Organization for Standardization (ISO) and the European Committee for Standardization (CEN), they have reached agreement on the standard definitions of inhalable, thoracic and respirable fractions of airborne particles inhaled and deposited in various regions of respiratory system [8]. Respirable dust fraction is defined as a fraction of the inhaled airborne particles likely to reach the gas exchange region of the lungs where removal mechanisms are less efficient compared to upper airways [8]. Examples of respirable dusts include quartz, hard metal dust, cobalt-containing and other dusts containing free crystalline silica [8]. The focus of this study is on this type of dust as it can penetrate deep into the alveoli and cause respiratory problems.

The threshold limit value (TLV) for long-term exposure in an eight-hour time weighted average reference period for respirable dust exposure is 5 mg/m^3^ [9]. Personal monitoring is used to establish the concentration of respirable dust within the breathing zone of an employee [8]. In order to protect workers from dust, control measures should be kept in place.

Many South African Development Community (SADC) countries, including Tanzania, have concentrated on managing work exposures by ensuring provision of personal protective equipment (PPE) to workers, which is actually the last line of defense [10]. Greater emphasis of control measures should follow the hierarchy: elimination, substitution, isolation, engineering controls, administrative control and PPE [11].

Miners are exposed to dust because the whole process of extracting gold has to involve rock breaking through blasting and drilling [12], as well as loading and unloading materials. That is why the International Labour Organization (ILO) came up with a guide on prevention and suppression of dust in mining [13]. But these measures of protection seem to be either not in place, inadequate or ineffectual, which is why the prevalence of respiratory symptoms among miners is still high [6]. In a study done in South Africa, the proportion of gold miners with silicosis has increased tenfold from 3% to 32% from 1975 to 2007 [14]. There is also significant evidence that shows an increasing effect of total cumulative dust exposure on breathlessness, which is a respiratory symptom [15].

Little is known about dust exposure in gold miners in Tanzania, even though gold accounts for 90% of the value of Tanzania mineral exports [2]. Even the research that has been done regarding dust in gold mines has mainly focused on either artisanal or small-scale miners who are expected to have low technology and not enough funds to implement proper control measures. This study aimed to focus on large-scale modern mining by studying respiratory problems and measuring the levels of exposure of personal respirable dust.

## Methodology

This was a cross-sectional study design that took place in a large-scale gold mine in Tanzania, with both underground and open pit operations. The study population consisted of miners who had worked in dusty areas for not less than one year during the time of the study and had given their consent to participate in the study. Workers who had chest surgery, recent abdominal surgery and those with heart diseases were excluded from the study.

The sample size for respiratory impairment was obtained through OpenEpi Version 3, open source calculator, while a National Institute for Occupational Safety and Health (NIOSH) sample size determination table [16] was used as a guideline to obtain a sample size for personal respirable dust exposure levels. There were 115 miners included in the study, including 4 similar-exposure groups (SEG) from underground operations and 4 similar-exposure groups from the open pit section. Half of the sample size for each similar-exposure group was repeated to account for within- and between-worker sources of variability [17]. A total of 140 samples for personal respirable dust were collected.

In the study, lung functioning tests indices and respiratory symptoms were dependent variables; whereas, age, duration of exposure, job category, level of education and working section were the independent variables.

Full shift personal dust monitoring was done using air sampling pumps (Air check XR 5000). Sampling heads (cassettes) were placed on the lapel at workers’ breathing zones. Air sampling pumps were calibrated before and after using dry calibrator (Defender 610), and flow rate was set at 2.0+/–0.1 litres/min. A questionnaire on respiratory symptoms adopted from the British council of medical research that had been pretested and used in Tanzania was used. Lung function tests were performed before and after working shifts using KoKo Legend S× 1000 Spirometer, nSpire Health. Values of forced vital capacity, forced expiratory volume in 1 second, and the ratio of forced expiratory volume in 1 second to forced vital capacity (FEV1/FVC) were recorded. The physician did a precalibration and performed the test on workers using forced expiratory maneuvers at least three times in standing position. The lung functioning tests were expressed as percentages of the expected values adjusted for age, body weight, height, sex and ethnicity [18]. Information on dust control measures was gathered using an observation checklist.

Field data were coded, cleaned and analysed using SPSS version 20, and the significance level was set at p < 0.05. An independent t-test was used to compare the arithmetic and geometric means of personal respirable dust exposure levels between similar exposure groups, with truck operators as a reference group for both underground and open pit miners. One-way analysis of variance (ANOVA) using the Bonferroni as a post-hoc test was used to compare mean differences of personal respirable dust within and between similar exposure groups in both underground and open pit sections. Fishers exact test and Chi-square tests were used to compare proportions of workers having an outcome variable (respiratory symptoms or respiratory impairment) and the independent variables, such as duration of employment, education level and working section. Multivariate logistic regression analysis was used to analyze how well a set of variables can predict an outcome. Duration of employment was categorized as short duration (1–5 years) and long duration (>5 years).

Ethical clearance was sought from Muhimbili University of Health and Allied Sciences Research and Publications Committee. Permission to conduct a study was asked from the gold mine administration. Each study participant was provided with an informed consent form. Those who were found to have a respiratory impairment were referred to the medical superintendent of the mine clinic for further management.

## Results

### Sociodemographic Characteristics

The results for sociodemographic characteristics are summarized in Table 1. This study had participants with a mean age of 37.4 years (SD = 6.4), ranging from 23 to 57 years. Males comprised 95.5% of participants. Among the study participants, 24.1% had primary education, 69.6% had secondary education and 6.2% had tertiary education. A majority of participants (69.6%) had been in employment for a short duration (1–5 years).

**Table 1:** Sociodemographic characteristics of study participants (n = 112).

Variable	Frequency	%

	n	%

**Age in group (years)**	
21–30	18	16.1
31–40	58	51.8
41–50	34	30.4
51–60	2	1.8
**Sex**	
Male	107	95.5
Female	5	4.5
**Education Level**	
Primary Education	27	24.1
Secondary Education	78	69.6
Tertiary Education	7	6.2
**Duration of Employment**	
1–5 years	78	69.6
Above 5 years	34	30.4
Current smokers	14	12.5
Ever smokers	6	5.4
Ever worked in a dusty job before	73	65.2
Ever exposed to gas or chemical fumes in previous job	4	3.6

Among the study participants (n = 112), 12.5% were smokers, 65.2% had worked in a dust job before, 3.6% had been exposed to gas or chemical fumes in previous jobs. The response rate was 97.4%.

### Personal Respirable Dust Exposure Levels among the Gold Miners

A total of 140 respirable dust samples were collected from 4 underground observational groups and 4 open cast observational groups. Arithmetic mean (AM) for respirable dust was 0.35 mg/m^3^ (SD = 0.28), ranging from 0.08 to 2.11 mg/m^3^ over a mean sampling time of 8 hours ranging between 7–11 hours. The overall geometric mean (GM) was 0.26 mg/m^3^ (GSD = 0.32). The GM for the underground group (0.41 ± 0.28 mg/m^3^) was significantly higher compared to the open pit group (0.17 ± 0.23 mg/m^3^), t (131.9) = 9.15, p < 0.01.

For the underground section, the GM was highest among the bogger operators at 0.53 mg/m^3^ (GSD = 0.28) and the least among the articulated dump truck operators at 0.29 mg/m^3^ (GSD = 0.36). For the open pit section, the GM was the highest among the quality controllers at 0.39 mg/m^3^ (GSD = 0.18) and the least among the truck operators at 0.13 mg/m^3^ (GSD = 0.15). These findings are summarized in Tables 2 and 4.

**Table 2:** Personal respirable dust exposure levels among similar exposure groups—Underground.

Similar Exposure Groups	Type of Activities	No. Samples	Mean Concentrations(mg/m^3^)			Range

			AM(SD)	GM(GSD)	Median	

Truck Operators	Haulage	19	0.38(0.24)	0.29(0.37)	0.45	0.08–0.80
Jumbo/Long Hole Drill Operators	Drilling	15	0.39(0.18)	0.36(0.20)	0.35	0.17–0.75
Bogger Operators	Loading materials	18	0.65(0.49)	0.53(0.28)	0.44	0.17–2.11
Offsiders	Assisting Jumbo	18	0.56(0.17)	0.52(0.15)	0.52	0.23–0.82

The independent t-test on log-transformed data comparing personal respirable dust exposure among the four underground exposure groups using truck operators as a reference group showed that offsiders had statistically significant higher personal respirable dust exposure levels (GM 0.52 ± 0.15 mg/m^3^) compared to truck operators (GM 0.29 ± 0.37 mg/m^3^), t (24.2) = –2.85, p = 0.009. The bogger operators had higher personal respirable dust exposure levels (GM 0.53 ± 0.28 mg/m^3^) compared to truck operators (GM 0.29 ± 0.37 mg/m^3^), t (33.15) = –2.46, p = 0.019. Jumbo/long hole driller operators had slightly higher personal respirable dust exposure levels (GM 0.36 ± 0.20 mg/m^3^) compared to truck operators (GM 0.29 ± 0.37 mg/m^3^), t (28.55) = –0.91, p = 0.37. The difference was not statistically significant as seen in Tables 3.

**Table 3:** Comparison of personal respirable dust exposure levels between similar exposure groups among underground gold miners.

Similar Exposure Groups	No. Samples	GM(GSD)	Range	95%CI of the difference	P-Value	

Truck Operators	19	0.29(0.37)	0.08–0.80	Reference		
Jumbo/Long Hole Drill Operators	15	0.36(0.20)	0.17–0.75	–0.29–0.11	0.37	
Bogger Operators	18	0.53(0.28)	0.17–2.11	–0.47–0.04	0.019	*
Offsiders	18	0.52(0.15)	0.23–0.82	–0.45–0.07	0.009	*

**Table 4:** Personal respirable dust exposure levels among similar exposure groups—Open pit.

Observational group	Type of Activities	No. Samples	Mean Concentrations(mg/m^3^)			Range

			AM(SD)	GM(GSD)	Median	

Truck Operators	Haulage	27	0.14(0.06)	0.13(0.15)	0.11	0.09–0.27
Excavator Operators	Loading materials	16	0.18(0.09)	0.16(0.21)	0.14	0.10–0.33
Dozer Operators	Clearance and pushing materials	18	0.17(0.08)	0.16(0.18)	0.16	0.10–0.32
Quality Controller	Measuring depth of holes	9	0.41(0.15)	0.39(0.18)	0.46	0.21–0.61

One way ANOVA with a post-hoc test (Bonferroni) was used to conduct further analysis of personal respirable dust exposure levels between the four similar exposure groups. There was a statistically significant difference (p < 0.05) in personal respirable dust levels between the four similar exposure groups [F (3,66) = 4.4, p = 0.007]. Post-hoc comparisons using the Bonferroni test indicated that the mean personal respirable dust concentrations for the offsiders (GM = 0.52, GSD = 0.15) was significantly different from the truck operators (GM = 0.29, GSD = 0.37), with p = 0.023. Moreover, the mean personal respirable dust concentrations of bogger operators (GM = 0.53, GSD = 0.28) was significantly different from the truck operators (GM = 0.29, GSD = 0.37), with p = 0.023.

The independent t-test on log-transformed data comparing personal respirable dust exposure levels among the four open-pit exposure groups using truck operators as a reference group showed that the quality controllers had higher personal respirable dust exposure levels (GM 0.39 ± 0.18 mg/m^3^) than the truck operators (GM 0.13 ± 0.15 mg/m^3^), t (34) = –7.67, p =< 0.01. The differences in respirable dust exposure levels between the excavators, dozers and truck operators was statistically insignificant as shown in Table 5.

**Table 5:** Comparison of personal respirable dust exposure levels between similar exposure groups among open pit gold miners.

Similar Exposure Groups	No. Samples	GM(GSD)	Range	95%CI of the difference	P-Value	

Truck Operators	27	0.13(0.15)	0.09–0.27	Reference		
Excavator Operators	16	0.16(0.21)	0.10–0.33	–0.20–0.45	0.21	
Dozer Operators	18	0.16(0.18)	0.10–0.32	–0.19–0.02	0.12	
Quality Controller	9	0.39(0.18)	0.21–0.61	–0.59–0.34	p < 0.001	*

Further analysis of personal respirable dust exposure levels was conducted between the four similar exposure groups in the open pit section using one-way ANOVA with a post-hoc test (Bonferroni). In this test, we established that the difference in personal respirable dust levels was statistically significant (at p < 0.05) between the four similar exposure groups [F (3, 66) = 15.6, p < 0.001]. Post-hoc comparisons using the Bonferroni test indicated that the mean respirable dust concentrations among the quality controllers (GM = 0.39, GSD = 0.18) was significantly higher compared to the truck operators (GM = 0.13 ± 0.15 mg/m^3^, p < 0.001), the dozer operators (GM = 0.16 ± 0.18 mg/m^3^, p < 0.001) and the excavator operators (GM = 0.16 ± 0.21 mg/m^3^, p < 0.001). There was no statistically significant difference in the mean respirable dust concentrations between the dozer and the excavator operators. Furthermore, the mean respirable dust concentrations among the truck operators significantly differed from the dozer and the excavator operators. The effect size calculated using eta squared was 0.7.

An independent t-test comparing respirable dust exposure levels between those who were in vehicles that had air conditioning (A/C) systems (enabling the closure of cabins) and those who were in vehicles that had no A/C (restricting the closure of the windows of their cabins) showed that those whose vehicles had no A/C had statistically higher exposure levels (GM 0.48 ± 0.20) compared to those who had A/C (GM 0.17 ± 0.29), t = 1.77, p < 0.001. The proportion of participants using vehicles with A/C was 33.9% for the underground workers and 87.1% for the open pit workers.

### The Prevalence of Respiratory Symptoms

**Figure 1:**
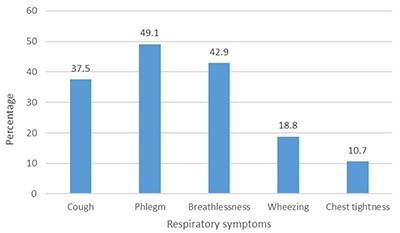
Prevalence of respiratory symptoms among Gold miners.

All the study participants (n = 112) were interviewed for respiratory symptoms. The most prevalent was phlegm (49.1%), followed by breathlessness (42.9%), cough (37.5%), wheezing (18.8%) and chest tightness (10.7%). Figure 1 depicts the proportion of respiratory symptoms among the studied gold mine workers.

### Previous Chest Illnesses among the Gold Miners

Previous chest illnesses among the studied gold miners stood at 2.7%. One of them had been diagnosed with emphysema and two of them with heart attack.

### Relationship between Respiratory Symptoms and Other Factors among the Gold Mine Workers

#### Working section

Respondents who worked at the underground section of the mine had higher symptoms of phlegm (62.0%) and wheezing (26.0%); whereas, respondents who worked at the open pit section of the mine had higher symptoms of cough (38.7%), breathlessness (45.2%) and chest tightness (12.9%). The difference for phlegm was statistically significant at p < 0.05 (see Table 6).

**Table 6:** Comparison of prevalence of respiratory symptoms between underground and open pit miners.

Respiratory Symptom	Working Section		*x*^2^	P value	

	Underground	Open pit			

Cough			0.010	0.922	
Yes	18(36.0)	24(38.7)			
No	32(64.0)	38(61.3)			
Phlegm			5.112	0.024	*
Yes	31(62.0)	24(38.7)			
No	19(38.0)	38(61.3)			
Breathlessness			0.127	0.721	
Yes	20(40.0)	28(45.2)			
No	30(60.0)	34(54.8)			
Wheezing			2.316	0.128	
Yes	13(26.0)	8(12.9)			
No	37(74.0)	54(87.1)			
Chest tightness			0.277	0.598	
Yes	4(8.0)	8(12.9)			
No	46(92.0)	54(87.1)			

Multivariate logistic regression analysis showed that there is an association between smoking and breathlessness. The participants who smoked were six times more likely to get breathlessness than those who did not smoke (see Table 7).

**Table 7:** Predictors of respiratory symptoms among gold cast miners.

Respiratory Symptoms	β	SE	OR(95%CI)	P value

**Cough**				
Duration of Employment	0.048	0.061	0.953(0.0846–1.073)	0.425
Cigarette smoking	–0.122	0.61	0.885(0.268–2.922)	0.841
Previous dust exposure	–0.009	0.46	0.991(0.402–2.441)	0.984
Age	–0.043	0.036	0.958(0.892–1.029)	0.237
**Phlegm**				
Duration of Exposure	–0.120	0.062	0.877(0.785–1.001)	0.053
Cigarette smoking	0.554	0.611	1.740(0.525–5.766)	0.365
Previous dust exposure	0.101	0.455	1.106(0.453–2.700)	0.825
Age	0.056	0.036	1.057(0.986–1.134)	0.119
**Breathlessness**				
Duration of Exposure	–0.058	0.063	0.944(0.835–1.067)	0.358
Cigarette smoking	1.849	0.700	6.353(1.611–25.052)	0.008*
Previous dust exposure	–0.232	0.474	0.793(0.313–2.006)	0.624
Age	0.048	0.037	1.049(0.977–1.128)	0.189
**Wheezing**				
Duration of Exposure	–0.115	0.090	0.891(0.747–1.063)	0.199
Cigarette smoking	0.382	0.756	1.465(0.333–6.446)	0.614
Previous dust exposure	1.469	0.816	4.343(0.878–21.447)	0.072
Age	0.068	0.047	1.070(0.977–1.173)	0.145
**Chest tightness**				
Duration of Exposure	0.123	0.092	1.135(0.947–1.360)	0.171
Cigarette smoking	0.635	1.117	0.530(0.059–4.729)	0.570
Previous dust exposure	1.440	0.913	4.222(0.705–25.280)	0.115
Age	0.020	0.057	1.021(0.914–1.140)	0.718

** Categorized as 1–5 years (short duration) and 6–15 years (long duration). * Logistic regression, odds ratio, 95%CI, P-value < 0.005.

#### Lung function impairment

**Figure 2:**
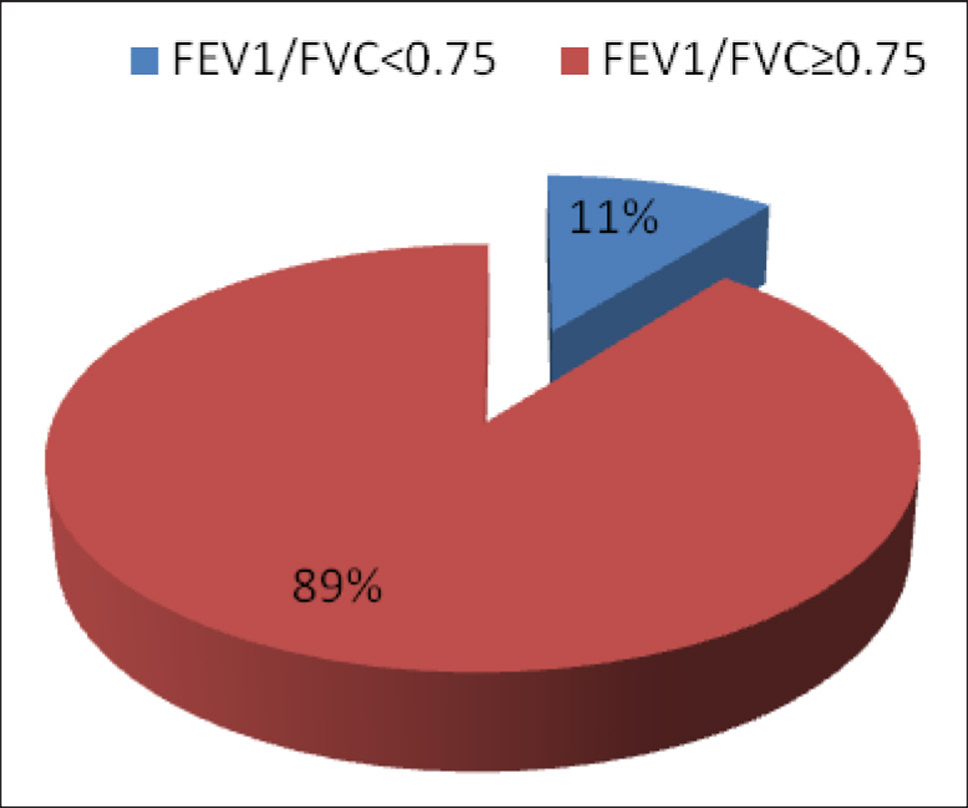
The prevalence of FEV1/FVC < 0.75.

A total of 104 out of 112 participants achieved acceptable curves. The remaining 8 (7.1%) could not produce any acceptable blows even after 8 consecutive maneuvers. The prevalence of lung function impairment was 10.6% for FEV1/FVC < 0.75 and 2 (1.9%) for FEV1/FVC < 0.7. Figure 2 presents the findings.

**Figure 3:**
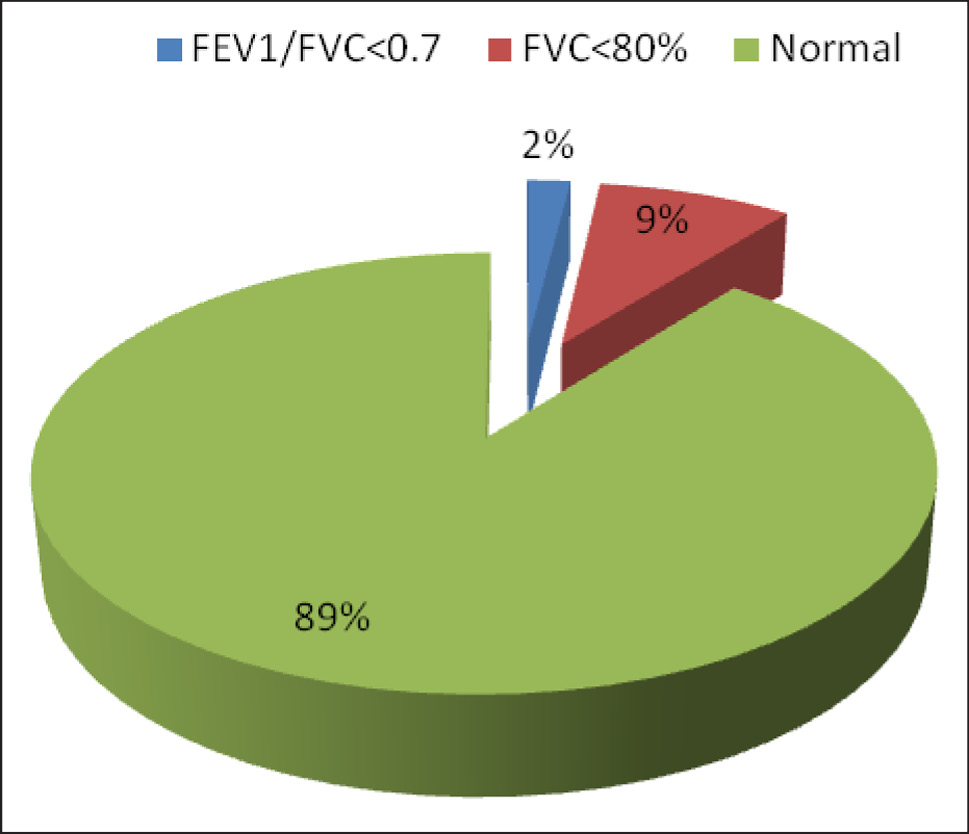
The prevalence of FEV1/FVC < 0.7 and FVC < 80%.

Among 102 participants that had FEV1/FVC ≥ 0.7, nine (8.8%) had FVC < 80%, which are lung restrictions. Figure 3 portrays the findings.

Age, cigarette smoking and previous dust exposure did not predict the outcome of lung function impairment as seen in Table 8.

**Table 8:** Predictors of lung function impairment among gold miners.

Lung function Impairment	β	SE	OR(95%CI)	P value

Age	0.000	0.053	1.000(0.902–1.109)	0.995
Cigarette smoking	–0.495	0.850	0.61(0.115–3.226)	0.561
Previous dust exposure	0.164	0.679	1.178(0.311–4.455)	0.810

#### Available Control Measures

In our survey, we observed that engineering control measures for open pit operations included heavy machine equipment with enclosed cab filtration systems and drill dust collection systems for drilling machines. In addition, there was a water truck that always sprayed water for controlling environmental dust. In the underground section of the mine, engineering control measures observed were the ventilation system and wetting agents for the roads outside the mine; water from drilled rocks kept the underground mine wet all the time. Conversely, most of the heavy equipment machines did not have air conditioning. As a result, the controllers of the machines could not close the windows of their cabins.

Administrative control measures observed were registered by the Occupational Safety and Health Administration (OSHA) and had a company OHS policy. In addition, they had written procedures for dust control, and respiratory protective equipment (RPE) were adequately available and easily accessed at their PPE warehouse. Moreover, OHS representatives were available in each department, and trainings on OHS issues, including dust control, were provided at least once a week for both underground and open pit operations. Dust masks were also provided to all workers at all working periods. Blasting was done by remote control at specific times of the day. We also observed that blasters ensured that all personnel had moved out of the mine before blasting rocks. There was also a schedule for one medical examination a year.

## Discussion

### Personal Respirable Dust Exposure Levels

The underground and open pit workers were both exposed to respirable dust below the TLV of the ACGIH for respirable dust of 5 mg/m^3^ [9] even though the mean respirable dust exposure level of underground mining was higher than that of the open pit mining [19]. The amount of respirable dust exposure of underground mining matches the 0.4 mg/m^3^ found at the underground gold mine of AngloGold Ashanti (Obuasi) Limited [20]. However, another study done among the underground gold miners in Obuasi-Ghana found that workers were exposed to the mean respirable dust concentration of 0.83 mg/m^3^—twice the amount found in this study [21].

Differences in the mean respirable exposure levels between the underground and open pit sections could have been caused by the underground mine being contained; hence, the workers require a local ventilation system compared to the open pit mine, where the natural air circulates freely. In addition, this study found there was a significant association between respirable dust exposure levels and the availability of air conditioning as an engineering control measure in enclosed cabins of heavy machine equipment [22].

We observed that gold mining activities, such as blasting, drilling, loading and unloading of materials, subjected workers to personal respirable dust at different levels according to their job type [23]. One way ANOVA showed a significant difference in GM of personal respirable dust exposure concentrations between and within SEGs. A post-hoc comparison using the Bonferroni test showed personal respirable dust exposure levels were significantly higher among workers dealing with underground drilling activities compared to those dealing with haulage activities. The findings are similar to those of a study done in Tanzania wherein respirable dust exposure measurements were significantly higher in those dealing with drilling activities than those dealing with loading and shoveling [23]. Personal respirable dust exposure among quality controllers was significantly higher compared to the truck, dozer and excavator operators. This is due to the heavy machine operators operating equipment with enclosed cabins and air conditioning systems [22]; whereas, the quality controllers work in direct contact with dust, and the only control measure they use is putting on dust masks, which is PPE and the last line of defense [11].

The study found that personal respirable dust exposure levels were relatively lower than the TLV recommended by the ACGIH. It is therefore probable that the rocks in the mines contain silica, which is known to cause silicosis, emphysema, chronic bronchitis, tuberculosis and renal disease [7]. Silica is a human carcinogen in the International Agency for Research on Cancer (IARC) group 1 [23], which means that it can cause cancer. However, testing the existence of silica in the rocks was beyond the scope of the study.

### Respiratory Symptoms among the Gold Miners

Despite the fact that personal respirable dust exposure levels were below the TLV recommended by the ACGIH, the prevalence of respiratory symptoms among the gold miners was still high: phlegm (49.1%), breathlessness (42.9%) and cough (37.5%). These findings are similar to those in a study done in AngloGold Ashanti (Obuasi) Limited, where cough was reported to be 28.5%, almost half of the respondents reported phlegm, while dyspnoea stood at 67%, irrespective of a time-weighted average of respirable dust being 0.4 mg/m^3^. In another study done in a coal mine in Tanzania, the prevalence of respiratory symptoms among miners was high [6]. This suggests the crystalline silica content was high, supporting a high prevalence of respiratory symptoms.

In this study, 2.7% reported ever being diagnosed with any of the stipulated chest illnesses. This is most likely because of the “healthy worker effect,” a phenomenon commonly observed in occupational studies wherein the severely ill and the most affected are ordinarily excluded from employment. This suggests that workers who have been highly affected by dust and suffered chronic chest illnesses were more likely not to be at work during the time of data collection [24]. Also the presence of a clinic that performs a periodic medical examination of the workers in the mine might have also contributed to the healthy worker effect. This was contrary to the findings of the study done among the Basotho gold miners; wherein almost 50% reported to have suffered either silicosis, past tuberculosis, current tuberculosis or chronic productive cough [25]. The same study done in South Africa had a high prevalence of current smokers 35% and ever smokers 61%, while the study in Ghana had 5.2% current smokers and 17.9% ever smokers [15]. Thus, when compared to this study, which had current smokers (12.5%) and ever smokers (6.1%), the likelihood of smoking contributing to the observed findings is very low.

There was no difference between underground and open pit workers in the proportion of workers suffering from most respiratory symptoms except for phlegm, where 62% of underground workers reported phlegm. The finding corroborates those reported among the underground miners in AngloGold Ashanti (Obuasi) Limited —almost half of the population was reported to have phlegm and 28.5% reported cough [20]. The similarity of the proportions among workers in these studies could be explained by the fact that a large proportion (65.2%) of the participants experienced dust exposure from previous jobs, and 89% of them had worked in a mining job before. A supposition is that they might have previously worked in an open pit mine before switching to an underground mine and vice versa. Be that as it may, the previous dust exposure levels were not quantified by this study. Hence, these assumptions remain speculations until confirmed by further studies.

In the present study, we did not find an association between the duration of employment and respiratory symptoms. Similarly, there was no association between duration of underground service and chronic bronchitis in Ghana; only an association between breathlessness grade and duration of underground service was reported [15]. It is also more likely that we did not find an association between the duration of employment and respiratory symptoms because only 30.4% of the workers had been employed there for more than 5 years. This is due to the fact that the gold mine started underground operations only 3 years ago, so for underground operations, the maximum duration of employment would be 3 years, which is inclusive in the category of short duration. The finding that the underground miners were more affected with respiratory symptoms than the open pit workers despite a short duration of employment gives a rationale for further studies on the relationship between exposure and outcome. Furthermore, the healthy worker effect [24], the nature of the dusts that workers had been previously exposed to and the high proportion of respondents who reported previous exposure to dust might have also affected the relationship between duration of employment and respiratory symptoms.

Workers who were current smokers were 6 times more likely to suffer from breathlessness compared to others, with statistical significance of p < 0.0008. These findings were similar to the ones found in Ghana and South Africa [15,26]. In Ghana, smoking was significantly associated with breathlessness grade, while in South Africa, it was found that smoking exacerbated the effects of dust on respiratory symptoms.

### Lung Function Impairment among the Gold Miners

The prevalence of FEV1/FVC < 0.7 and FEV1/FVC < 0.75 stood at 1.9% and 10.6%, respectively. These percentages were lower than those found among the coal miners in Tanzania, which was 17.1% for FEV1/FVC < 0.7. Similarly, they were lower than those found among the Basotho gold miners in South Africa: 13.4% and 26.3% for FEV1/FVC < 0.7 and FEV1/FVC < 0.75, respectively [25].

The prevalence of FVC < 80% in this study was found to be 8.8%. These results could be explained by the healthy worker effect. In addition, the fact that the mine owned a clinic that conducts preentry, periodic and exit medical examinations could have screened out workers with lung function impairments.

Age, cigarette smoking and previous exposure to dust did not predict an outcome of lung function impairment in this study. These results were contrary to the studies found in Ghana and South Africa [15,26,27,28,29]. These results could be supported by the healthy worker effect [24]. This effect could also support the lack of a prediction of lung function impairment by cigarette smoking because those whose respiratory systems are affected by either respirable dust, cigarette smoking or both could have been screened out during periodic medical examinations.

### Available Control Measures

In large-scale mines, operators of heavy machines are the ones who are often exposed to dust [22]. These operators were captured among the similar exposure groups included in this study, but engineering controls, such as enclosed cabins, enclosed cab filtration systems and drill dust collection systems were in place [22]. In addition, the presence of vehicles spraying water in the open pit mine and around both mines for underground operations and the water from the rocks in the underground section of the mine explain the relatively low respirable dust exposure levels obtained in this study. This is based on the fact that air and water systems alone have been shown to reduce up to 80% of dust concentrations [30]. The results are similar to studies in Chinese and Australian underground mines where a commonly found practice was the use of water during various mining activities [31].

The presence of administrative control measures, such as standard operating procedures (SOPs), training, the removal of personnel, periodic medical surveillance and the availability of respiratory protective equipment (RPE) also impacted the study’s outcome. This showed an employer commitment to protecting the health of employees [32]. RPEs are the last line of defense as a dust controlling measure [11]. There was a PPE warehouse at the mine where workers could access RPEs using their identity cards. Trainings on proper use of PPE were provided as one of the topics among those provided by the OHS department. However, utilization of RPEs was covered in our study.

## Conclusion and Recommendations

In this study we found a high prevalence of respiratory symptoms despite levels of personal respirable dust exposure for both underground and open pit operations below the TLV recommended by the ACGIH. We recommend follow-up studies to establish a causal-effect relationship and to remove the Hawthorne effect. Similarly, we recommend that studies on ex-miners be conducted to establish health effects emanating from hazards such as dust by controlling for the healthy worker effect. Further, we recommend that all heavy machine equipment be fitted with air conditioning systems to enable their operators to close cabin windows to reduce dust. In addition, the mine should conduct awareness campaigns on the effects of cigarette smoking on the respiratory system.
